# Commonly used intravenous anesthetics decrease bladder contractility: An *in vitro* study of the effects of propofol, ketamine, and midazolam on the rat bladder

**DOI:** 10.4103/0970-1591.70570

**Published:** 2010

**Authors:** Canan Ceran, Arzu Pampal, Ozgur Goktas, H. Kutluk Pampal, Ercument Olmez

**Affiliations:** Department of Pediatric Surgery, Inonu University, Malatya; 1 Department of Pediatric Surgery, Ufuk University, Ankara; 2 Department of Pharmacology, Inonu University, Malatya; 3 Department of Anesthesiology and Reanimation, Mesa Hospital, Ankara; 4 Department of Pharmacology, Inonu University, Malatya

**Keywords:** Bladder, smooth muscle contractility, propofol, ketamine, midazolam

## Abstract

**Aim:**

This study was designed to test the hypothesis that propofol, ketamine, and midazolam could alter the contractile activity of detrusor smooth muscle.

**Materials and Methods:**

Four detrusor muscle strips isolated from each rat bladder (n = 12) were placed in 4 tissue baths containing Krebs-Henseleit solution. The carbachol (10 ^−8^to 10^−4^mol/L)-induced contractile responses as well as 5, 10, 20, 30, 40, 50 Hz electrical field stimulation (EFS)-evoked contractile responses of the detrusor muscles were recorded using isometric contraction measurements. After obtaining basal responses, the *in vitro* effects of propofol, ketamine, midazolam (10^−5^ to 10^−3^ mol/L), and saline on the contractile responses of the detrusor muscle strips were recorded and evaluated.

**Results:**

All the 3 drugs reduced the carbachol-induced and/or EFS-evoked contractile responses of rat detrusor smooth muscles in different degrees. Midazolam (10^−4^ to 10^−3^ mol/L) caused a significant decrease in the contractile responses elicited by either EFS or carbachol (*P*=0.000−0.013). Propofol (10^−3^mol/L) caused a decrease only in EFS-evoked contractile responses (*P*=0.001−0.004) and ketamine (10^−3^mol/L) caused a decrease only in carbachol-induced contractile responses (*P*=0.001−0.034).

**Conclusion:**

We evaluated the effects of the 3 different intravenous anesthetics on detrusor contractile responses *in vitro* and found that there are possible interactions between anesthetic agents and detrusor contractile activity. The depressant effects of midazolam on the contractile activity were found to be more significant than ketamine and propofol. Despite the necessity of further studies, it could be a piece of wise advice to clinicians to keep the probable alterations due to intravenous anesthetics in mind, while evaluating the results of urodynamic studies in children under sedation.

## INTRODUCTION

Urodynamic study is a matter of choice in the clinical practice to evaluate not only the functional disorders of the lower urinary tract but also the coordination between detrusor muscle and sphincter. It is generally difficult to perform in children, and sedation is sometimes needed during this evaluation in children.

As most of the diagnostic and endoscopic procedures are performed under sedation, the interactions between the anesthetic drugs and their effects on the organs and systems have gained importance. Many of the intravenous anesthetics are known to relax the smooth muscles and/or decrease the contractile responses of the smooth muscles of various organs.[1–11] However, there is limited data in the literature about the effects of intravenous anesthetics on the contractile activity of the bladder smooth muscle.[12–14] Despite the widespread usage of the anesthetics during urodynamic studies in clinical practice, it is probable that intravenous anesthetics could affect the contractile activity of detrusor smooth muscle and could interfere with the results obtained from the patients.

This study is undertaken to determine the probable effects of commonly used 3 intravenous anesthetics (propofol, ketamine, and midazolam) on rat detrusor smooth muscle contractile responses.

## MATERIALS AND METHODS

The study protocol was approved by the Animal Ethics Committee and performed according to the guidelines of the Research Committee of Faculty of Medicine at Inonu University. All animals received human care in compliance with the “Principles of Laboratory Animal Care” formulated by National Society for Medical Research and the “Guide for the Care and Use of Laboratory Animals” prepared by the Institute of Laboratory Animal Resources published by the National Institutes of Health. Twelve young male Wistar albino rats weighing 250–300 gr were killed by a sharp blow to the head, which was followed by exsanguinations. The whole bladder was excised and placed in a plate containing oxygenated Krebs–Henseleit solution (mmol/L: NaCl 118, KCl 4.7, CaCl_2_ 2.5, MgSO_4_ 1.2, KH_2_PO_4_ 1.2, NaHCO_3_ 25, glucose 10, pH 7.4). Four detrusor muscle strips (2 × 8 mm) were obtained from each animal after cutting the bladder longitudinally. Each strip was only used to investigate the action of one sedative on a single type of external stimulus and then discarded. Six animals were used to test the effects of drugs on the EFS-evoked contractions and 6 were used to test the effects on carbachol-induced contractions.

Bladder strips were mounted in 4 vertical chambers of 20 mL capacity, containing Krebs–Henseleit solution and constantly oxygenated with a mixture of 95% O_2_ and 5% CO_2_. The lower end of the muscle strip was tied to a glass hook attached to the muscle-holder and the upper end was attached to a Grass force transducer (FT03, Grass Instruments, Quincy, MA), which was connected to an amplifier (P-122, Grass). The analog signal obtained from the force transducer was converted to a digital signal (Polyview, Grass) at a sampling rate of 5 Hz and stored on a computer in ASCII format.

Before starting the experiments, the strips were allowed to equilibrate under a passive resting tension of 1 gr for 60 min, during which the bath solution was changed every 10 min. At the beginning of each experiment, 80 mmol/L KCl was added to the tissue bath and the contraction was considered as the reference response (RR). At the end of each experiment, the contractile response to 80 mmol/L KCl was remeasured to verify that the contractile mechanisms are intact.

### Evaluating the effects of intravenous anesthetics on electrical field stimulation-evoked detrusor contractions

Following the first KCl response, tissues were washed twice and after a period of 30 min, electrical field stimulation (EFS), using Harward 50-8952 model stimulator, was accomplished by means of 2 platinum plate electrodes, positioned on either side of the tissue to activate the intrinsic nerves (n = 6). Each stimulation lasted 10 s at constant parameters: 50 V, 5 ms pulse duration, and the frequency had been increased gradually (5, 10, 20, 30, 40, 50 Hz) every 2 min. In separate series of experiments, EFS was repeated in the presence of ketamine, propofol, and midazolam at concentrations of 10^−5^ to 10^−3^mol/L in 3 of the baths and 1 bath served as control.

### Evaluating the effects of intravenous anesthetics on carbachol-induced detrusor contractions

Tissues were washed twice in a period of 30 min following the first KCl response, then the concentration–response curves to carbachol (10^−8^ to 10^−4^ mol/L) were obtained cumulatively (n = 6). Ketamine, propofol, and midazolam were applied (10^−5^ to 10^−3^ mol/L) in 3 of the baths and 1 bath served as control. Between each dose of the drugs, the tissues were washed twice and after a period of 30 min, the concentration#x2013;response curves to carbachol (10^−8^ to 10^−4^ mol/L) were obtained in the presence of each dose of ketamine, propofol, and midazolam again.

### Data analysis and statistics

Results are expressed as the percentages of the maximal contractions induced by first train of EFS in the EFS group and the maximal contractions induced by KCl (RR) in the carbachol group. The data were presented as the arithmetic mean ± SEM of the number (n) of experiments. Statistical analyses were performed using one-way analysis of variance (ANOVA) followed by Tukey multiple comparisons test with commercially available software (SPSS 10.0, Chicago, Illinois, USA). Values of *P* < 0.05 were considered to be statistically significant.

## RESULTS

### KCl responses

No statistically significant difference was observed in KCl responses before and after each experiment [Table 1].

**Table 1:** The contractile responses to KCl

Bath number	Before experiment		After experiment	
	Mean	SD	Mean	SD
1	8265.3	993.2	8172.6	1543.8
2	7936.3	1224.1	8246.5	1479
3	8402.6	1628.7	8211.1	1500.2
4	8823.3	1686.2	8797.3	1154.1
Total	8356.9	1356.7	8356.9	1356.7

### Effect of intravenous anesthetics on electrical field stimulation-evoked detrusor contractions

**Figure 1:**
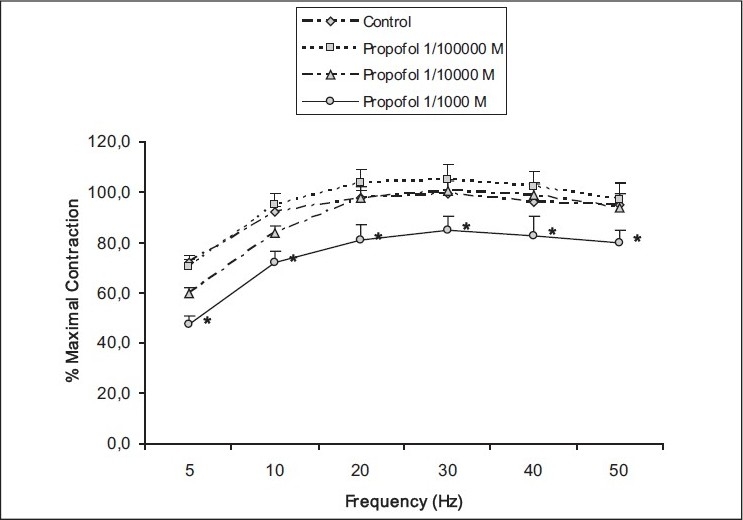
The effect of propofol (10^−5^ to 10^−3^ mol/L) on the electrical field stimulation–evoked contractile responses of the rat detrusor smooth muscle. Results represent the mean ± SEM of 6 experiments in each group. *P < 0.05 denotes significant difference from control.

High concentration of propofol (10^−3^ mol/L) caused a significant decrease in the contractile responses evoked by EFS at all frequencies, whereas no significant effect was determined for lower concentrations (*P* = 0.001–0.004) [Figure 1]. As an example, the maximal contractile response evoked by EFS at 5 Hz was found elevated at a high concentration of propofol (10^−3^ mol/L) when compared with saline administration (71.8 ± 5.7 vs 47.2 ± 11.8% of maximal contraction, *P* < 0.003).

Ketamin did not significantly change the contractile responses evoked by EFS at concentrations of 10^−5^ to 10^−3^ mol/L when compared with the control values.

**Figure 2:**
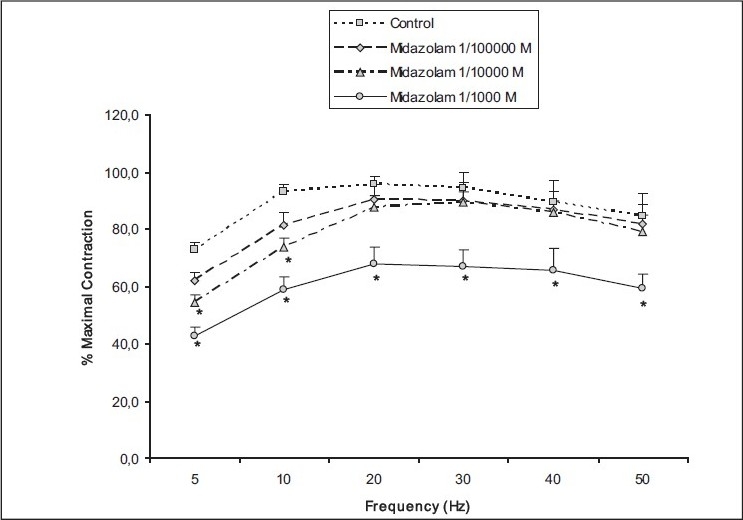
The effect of midazolam (10^−5^ to 10^−3^ mol/L) on the electrical field stimulation– evoked contractile responses of the rat detrusor smooth muscle. Results represent the mean ± SEM of 6 experiments in each group. *P < 0.05 denotes significant difference from control.

High concentration of midazolam (10^−3^ mol/L) in the bath reduced the contraction responses of rat detrusor smooth muscle evoked by all frequencies of EFS when compared with the control values (*P* = 0.000–0.013), whereas moderate concentration of midazolam (10^−4^ mol/L) reduced the contractile responses to EFS significantly only at low frequencies (5 and 10 Hz) [Figure 2]. The maximal contractile response evoked by EFS at 5 Hz was found to be decreased at a moderate concentration of midazolam (10^−4^ mol/L) when compared with saline administration (54.3 ± 6.5 vs 72.7 ± 7.4% of maximal contraction, *P* < 0.002).

### Effect of intravenous anesthetics on carbachol-induced detrusor contractions

Propofol did not significantly change the contractile responses induced by carbachol at concentrations of 10^−5^ to 10^−3^ mol/L when compared with the control values.

**Figure 3:**
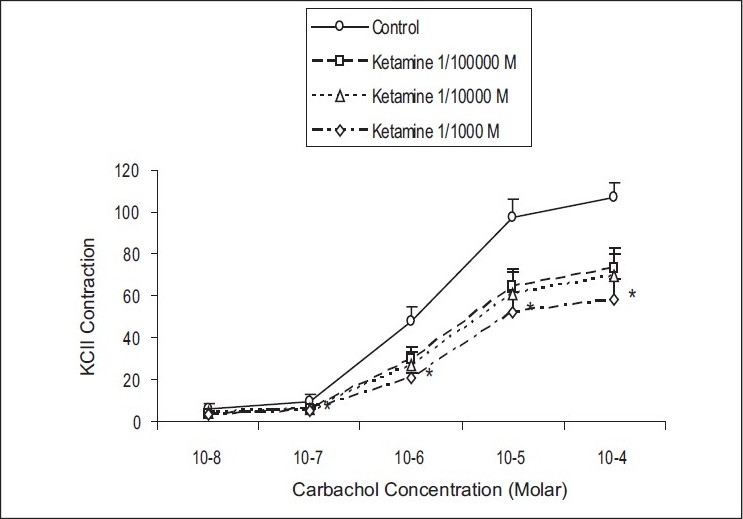
The effect of ketamine (10^−5^ to 10^−3^ mol/L) on the carbachol-induced contractile responses of the rat detrusor smooth muscle. Results represent the mean ± SEM of 6 experiments in each group. *P < 0.05 denotes significant difference from control.

High concentration of ketamin (10^−3^ mol/L) caused significant decrease in the contractile responses induced by carbachol at 10^−7^ and 10^−6^ mol/L concentrations (*P*=0.001–0.034) [Figure 3]. The addition of 10^−3^ mol/L ketamin to the organ baths caused decreases in carbachol-induced contractile responses from 13.3 ± 4.2 to 4.6±2.2% of maximal contraction at 10^−7^ mol/L carbachol (*P* < 0.001), and from 57.8 ± 9.2 to 23.8±5.1% of maximal contraction at 10^−6^ mol/L carbachol (*P* < 0.034).

**Figure 4:**
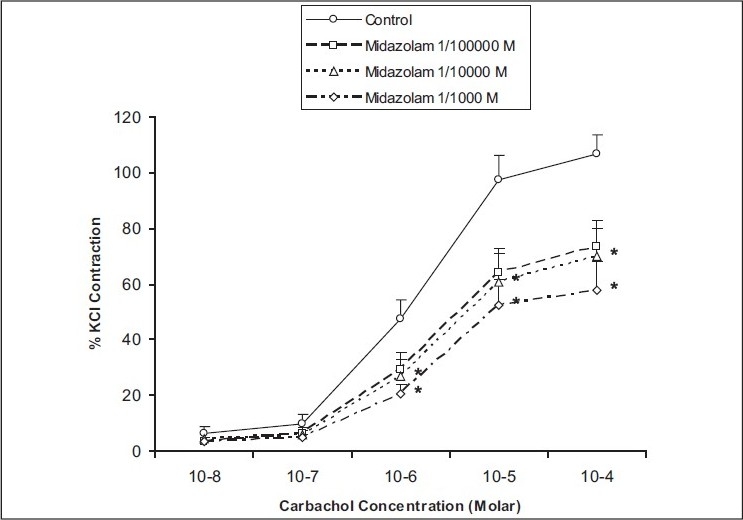
The effect of midazolam (10^−5^ to 10^−3^ mol/L) on the carbachol-induced contractile responses of the rat detrusor smooth muscle. Results represent the mean ± SEM of 6 experiments in each group. *P<0.05 denotes significant difference from control.

Moderate to high concentrations of midazolam (10^−4^ to 10^−3^ mol/L) reduced the carbachol-induced contractile responses of rat detrusor smooth muscle cumulatively, with significant decreases between 10^−6^ and 10^−4^ mol/L concentrations of carbachol (*P* = 0.006–0.046) [Figure 4]. The maximal contraction induced by 10^-6^ mol/L carbachol (47.5 ± 7.2%) was found to be decreased to 27.1 ± 3.6 and 20.6 ± 7.7% after addition of 10^−4^ mol/L and 10^−3^ mol/L midazolam to the organ baths, respectively (*P* < 0.015). The maximal contraction induced by 10^−5^ mol/L carbachol (97.5 ± 11.3%) was found to be decreased to 60.5 ± 7.2 and 52.1 ± 13.1% after addition of 10^−4^ and 10^−3^ mol/L midazolam to the organ baths, respectively (*P* < 0.015). The maximal contraction induced by 10^−4^ mol/L carbachol (107.2 ± 21.3%) was found to be decreased to 70.0 ± 24.2 and 57.8 ± 24.8% after addition of 10^−4^ and 10^−3^ mol/L midazolam to the organ baths, respectively (*P* < 0.015).

## DISCUSSION

This study investigated the *in vitro* effects of 3 intravenous anesthetics, midazolam, ketamine, and propofol, on nerve-mediated and carbachol-induced contractions of rat detrusor smooth muscles. Although all the 3 drugs reduced the contractile responses of the rat detrusor smooth muscle elicited by EFS and/or carbachol in different degrees, some of these results were not statistically significant. It was observed that 10^−3^ mol/L midazolam reduced EFS-evoked contractile responses at 5, 10, 20, 30, 40, and 50 Hz and 10^−4^ mol/L midazolam reduced EFS-evoked contractile responses elicited at 5 and 10 Hz. Midazolam (10^−4^ to 10^−3^ mol/L) also reduced carbachol-induced contractile responses at 10^−8^ to 10^−4^ mol/L concentrations significantly. Propofol at a concentration of 10^−3^ mol/L has significantly reduced EFS-evoked contractile responses but not the carbachol-induced ones. In contrast, 10^−3^ mol/L ketamin has significantly decreased the carbachol-induced contractile responses, but not the EFS-evoked ones.

Urodynamic studies of the lower urinary tract provide useful clinical information about the function of the urinary bladder, the sphincter mechanism, and the voiding pattern itself. As the application is invasive and uncomfortable, sedation is sometimes needed, especially in infants and toddlers. Relatively short-acting anesthetic agents, such as propofol, ketamine, and midazolam, are matter of choice for such interventions. However, there is very limited data about the effects of these agents on the bladder contractility. In order to evaluate these effects, an *in vitro* study with limited number of animals was performed.

The contractile responses gained from the application of EFS to a muscular strip give information about the whole neuromuscular junction (NMJ), including presynaptic and postsynaptic area. Application of exogenous carbachol, a cholinergic agonist, causes a contractile response via postsynaptic muscarinic receptors. Therefore, an appropriate response to the exogenous cholinergic agonist advocates a vigorous postsynaptic area.[15] In this study, we applied EFS and carbachol to the muscular strips in order to evaluate the contractile activity of detrusor muscle and KCl in order to consider the reference response.

Propofol, ketamine, and midazolam are commonly used anesthetic agents for sedation during diagnostic or endoscopic procedures. Their usage is advantageous because they have not only short action duration but also rapid onset of action.[16–18] Besides their well-known central nervous system depressant effects, there is limited data in the literature about the effects of these anesthetic agents on the autonomic nervous system.

As an anesthetic agent, propofol is known to produce parasympathetic predominance in the body. Its relaxant effects on tracheal smooth muscle, gastric or colonic muscle, pregnant uterine muscles, and diaphragm have been presented in the literature. Mills *et al*. found that propofol decreased the bladder compliance in pigs and atropine antagonized this action.[14] They concluded that the decrease in compliance seen in the pig bladder after sedation with propofol is mediated via muscarinic excitation. This study showed that a high concentration of propofol (10^−3^ mol/L) reduced EFS-evoked contractile responses significantly. But no difference on contractile responses was observed during carbachol-induced contractions. Further radioligand studies are needed in order to confirm these changes on the molecular aspects and to prove the underlying mechanism.

Ketamine is known to have sympathoexcitatory effects with unexplained action mechanisms. The effects of the drug are mediated by N-methyl-d-aspartate, opioid, muscarinic, and different voltage-gated receptors.[17] Moreover, its direct effects on both α_1_- and β_2_-adrenoreceptors of the urinary bladder and urethra were demonstrated by Bevan *et al*.[21] In our study, we found that 10^−3^ mol/L ketamine reduced carbachol-induced contractile responses significantly, but did not affect the contractile responses elicited by EFS.

Midazolam decreases tonic sympathetic activity.[18] Its depressant effects on CaCl_2_, KCl, and Ach-induced contractions of the urinary bladder were demonstrated by Marti-Cabrera *et al*.[12] Also, Symth *et al*. demonstrated the presence of peripheral-type benzodiazepine receptors in the urinary bladder, which are capable of altering contractility by the modulation of Ca^+2^ activity *in vitro*.[13] In this study, we found that a benzodiazepine derivative, midazolam at moderate to high concentrations (10^−4^ to 10^−3^ mol/L) reduced both EFS-evoked and carbachol-induced contractile responses significantly.

In an earlier study, Ghoniem *et al*. performed a controlled study comparing the effects of 2 anesthetics (flurane and ketamine) on urodynamics in a primate model and found that anesthesia has a profound effect on the bladder functions and capacity.[23] They proposed ketamine as a suitable agent for urodynamic studies in nonhuman primates. Being comparable with this study, we too suggest ketamine for urodynamic studies as it has less depressant effects on detrusor contractility when compared with the other anesthetic agents.

Bozkurt *et al*. investigated the effects of midazolam on urodynamics in children and reported that midazolam administration has not changed the results.[24] In our study, we demonstrated that moderate to high concentrations of midazolam reduced detrusor contractility significantly. This diversity can be attributed to the heterogeneity of the clinical study group presented by Bozkurt *et al*. They have performed this study on 20 patients, most of them having anorectal malformations and the children with anorectal malformations might have defective bladder functions due to either primary pathology or secondary to the operations.

Plasma levels needed for hypnosis and amnesia in humans are approximately 100–200 ng/mL for midazolam, 85–160 ng/mL for ketamine, and 2–6 μg/mL for propofol.[22] When the fact that these drugs are significantly protein-bound is also taken into account, it is almost certain that the concentrations used in this study are relevant to clinical practice.

The limitation of this study was the lack of application of receptor blockers, such as tetrodotoxin to the organ baths, while evaluating the effects of anesthetics. A further study evaluating such interactions would probably give more detailed information about the effects of the anesthetics at the NMJ and the type of the contractile response due to either nerve stimulation or muscle depolarization.

In conclusion, we demonstrated that the anesthetic agents, especially midazolam, depress the *in vitro* detrusor contractions significantly. These results bring to focus the effects of anesthetic agents on urodynamics. Further studies evaluating the effects of agonists and antagonists on the contractile responses in detrusor muscle under sedation, the urodynamic changes in human beings under sedation, or the effects of titration of the anesthetic agents used during urodynamic studies are needed to define the underlying mechanisms of the inhibitory effects of anesthetic agents on the detrusor muscle. We also think that the clinicians should bear in mind the probable alterations due to intravenous anesthetics, while evaluating the results of urodynamic studies in children under sedation.
